# Supplementary material for: U.S. regional differences in physical distancing: Evaluating racial and socioeconomic divides during the COVID-19 pandemic
Source: PLoS One. 2021 Nov 30;16(11):e0259665. doi: 10.1371/journal.pone.0259665 (PMC8631641; doi:10.1371/journal.pone.0259665)
Supplement: S1 Appendix — (DOCX) [file pone.0259665.s001.docx]

**S1 Appendix.**

In S1 Fig, we show several alternate measures of physical distancing to the 7-day rolling average of the proportion staying at home, which is the main metric used in our paper. These measures include the median proportion of devices staying home; the difference in stay-at-home proportions between a given date in 2020 and in 2019; the median time spent away from home; and the proportion of devices exhibiting “work behavior,” or spending more than three hours away from home between 8 a.m. and 6 p.m. local time (variable documentation online^1^).

We firstly show the median proportion of devices staying at home. Given a few block groups in SafeGraph’s location data include a disproportionately large number of cell phones for their population, we may overrepresent these few block groups by using the 7-day rolling mean proportion of devices staying home weighted by the total number of devices in each block group. Using the median proportion of devices staying home in each Census region can mitigate this issue. Secondly, we show the proportion staying completely at home relative to the same period last year, to show that our results are consistent in not only trends of stay-at-home patterns but also changing stay-at-home behavior in response to the lockdown orders. Thirdly, we show the median time away from home in minutes. The proportion staying completely at home may exclude those leaving their home for short excursions such as walks or performing necessary activities like grocery shopping. Therefore, it may underestimate the extent of physical distancing. The median time traveled can be an alternate measure distinguishing those that are exposed for longer periods of time and potentially experience higher risks from those who travel only short amounts of time immediately outside of their home. Finally, we show trends in the proportion of devices exhibiting “work behavior.” A device exhibiting “work behavior” is defined as any device that spends more than 3 hours at a location outside of their home during 8 a.m. and 6 p.m. local time or stops for more than 20 minutes at more than three locations outside of their geohash-7 home (characterized by SafeGraph as “delivery behavior”). For the median distance traveled from home and the work behavior metrics, we exclude weekends as a smoothing measure for our plots.

The alternative measures of physical distancing shown here are on the whole reflective of results from Fig 1. There is a sharp increase in physical distancing through April across all regions, with the greatest rates of physical distancing at the height of the pandemic across all metrics occurring in the Northeast, and physical distancing trends converging and more broadly lowering as the lockdown goes on.

Notably, define frontline workers as those occupations with the highest risks for sickness-related absences from work in March – June 2020. Specifically, existing studies show that sickness-related absences were highly concentrated in the following occupations: service, transportation, production, and material moving occupations ^2^. Our detailed coding of the frontline occupations is shown below, based on information provided in Lyttelton and Zang (2020).

S5 Fig shows our expanded definition of frontline workers, which includes all occupations in our primary definition of “frontline worker” and in addition healthcare, sales, and maintenance and repair occupations. Our results here are nearly identical to the results shown in Fig 4 with the primary definition of frontline workers, with trends in the Midwest, South, and West suggesting that frontline workers are not able to stay home at the height of the lockdown. This trend is reversed in the Northeast, when differences at the height of the lockdown are small but increase as the lockdown goes on.

Results in S3 and S4 Figs by Census division and state further show considerable variations within and across Census divisions. We observe largest within-region variations in the South Census region—namely, between the East-South-Central and South-Atlantic Census divisions, which maintained differences in stay-at-home rates of about 0.05 throughout the pandemic despite being indistinguishable before March 2020. Between the East-South-Central (South) and the Middle-Atlantic (Northeast) divisions, the difference is even more pronounced, with a 0.16 difference in stay-at-home rates at the beginning of April. Census divisions also differ by the states within them: while the four states in the West-North-Central division are nearly indistinguishable in their stay-at-home rates, much heterogeneity is present in the South-Atlantic division, with a 0.15 difference between Maryland and South Carolina at the beginning of April.

**Reference**

1. SafeGraph. Social Distancing Metrics. 2020; <https://docs.safegraph.com/docs/social-distancing-metrics>.

2. Lyttelton T, Zang E. Sickness-related Absences During the COVID-19 Pandemic: The Role of Occupations. 2020.
