# Supplementary figures and images for: U.S. regional differences in physical distancing: Evaluating racial and socioeconomic divides during the COVID-19 pandemic
Source: PLoS One. 2021 Nov 30;16(11):e0259665. doi: 10.1371/journal.pone.0259665 (PMC8631641; doi:10.1371/journal.pone.0259665)

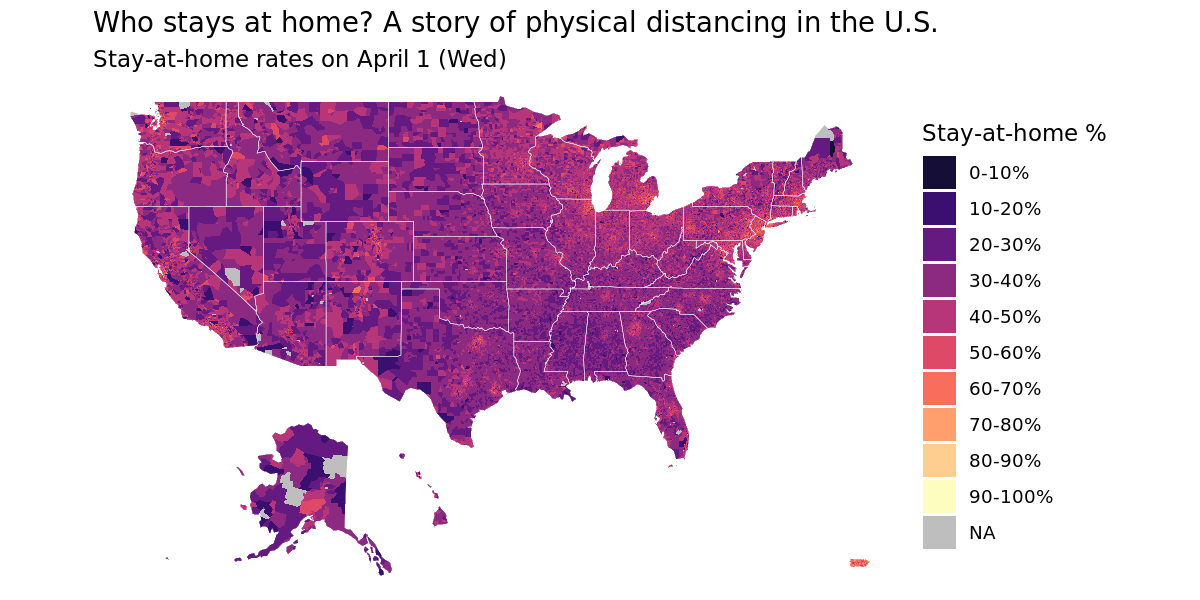

Supplement: S1 Fig — (PNG) [file pone.0259665.s002.png]

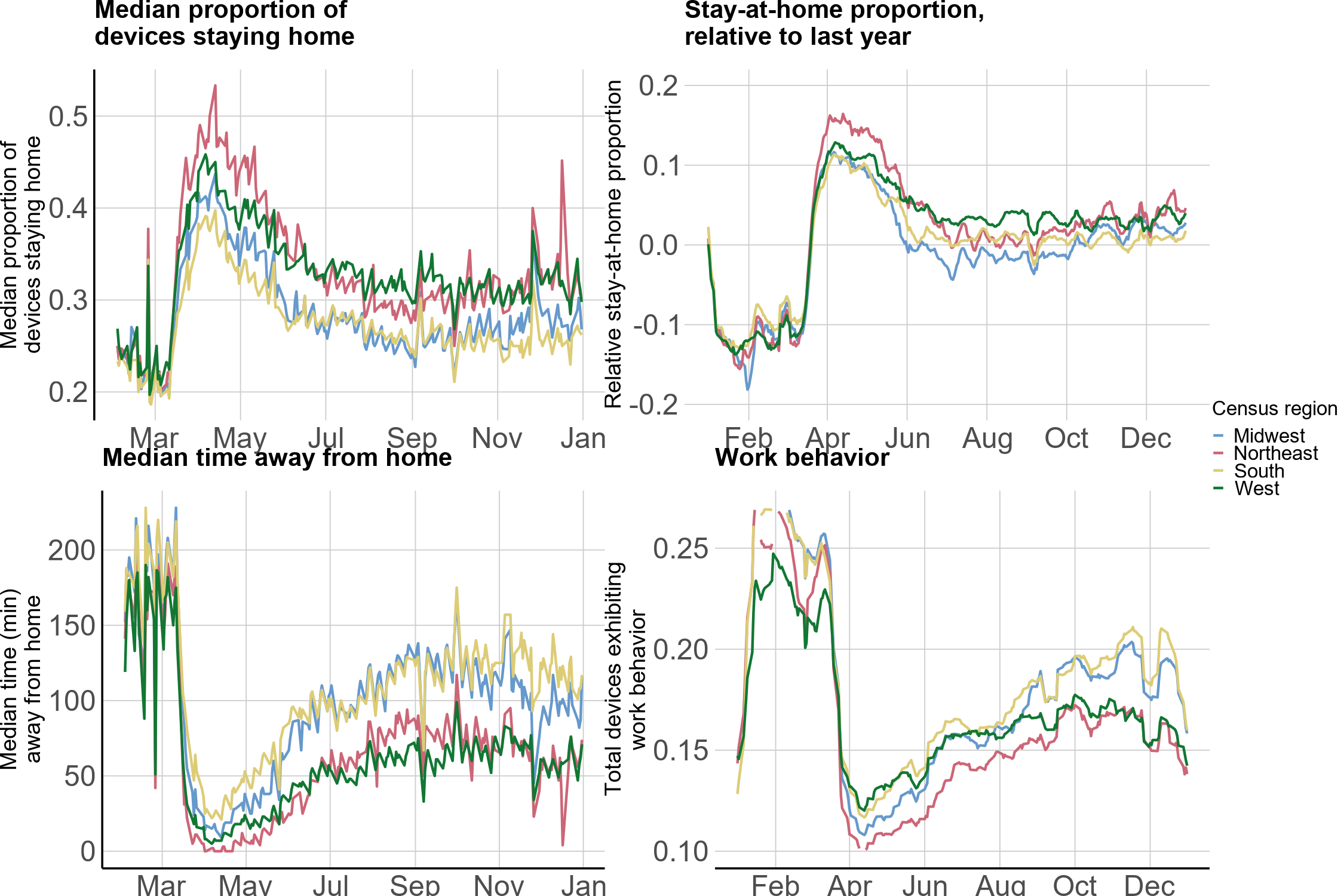

Supplement: S2 Fig — (TIFF) [file pone.0259665.s003.tiff]

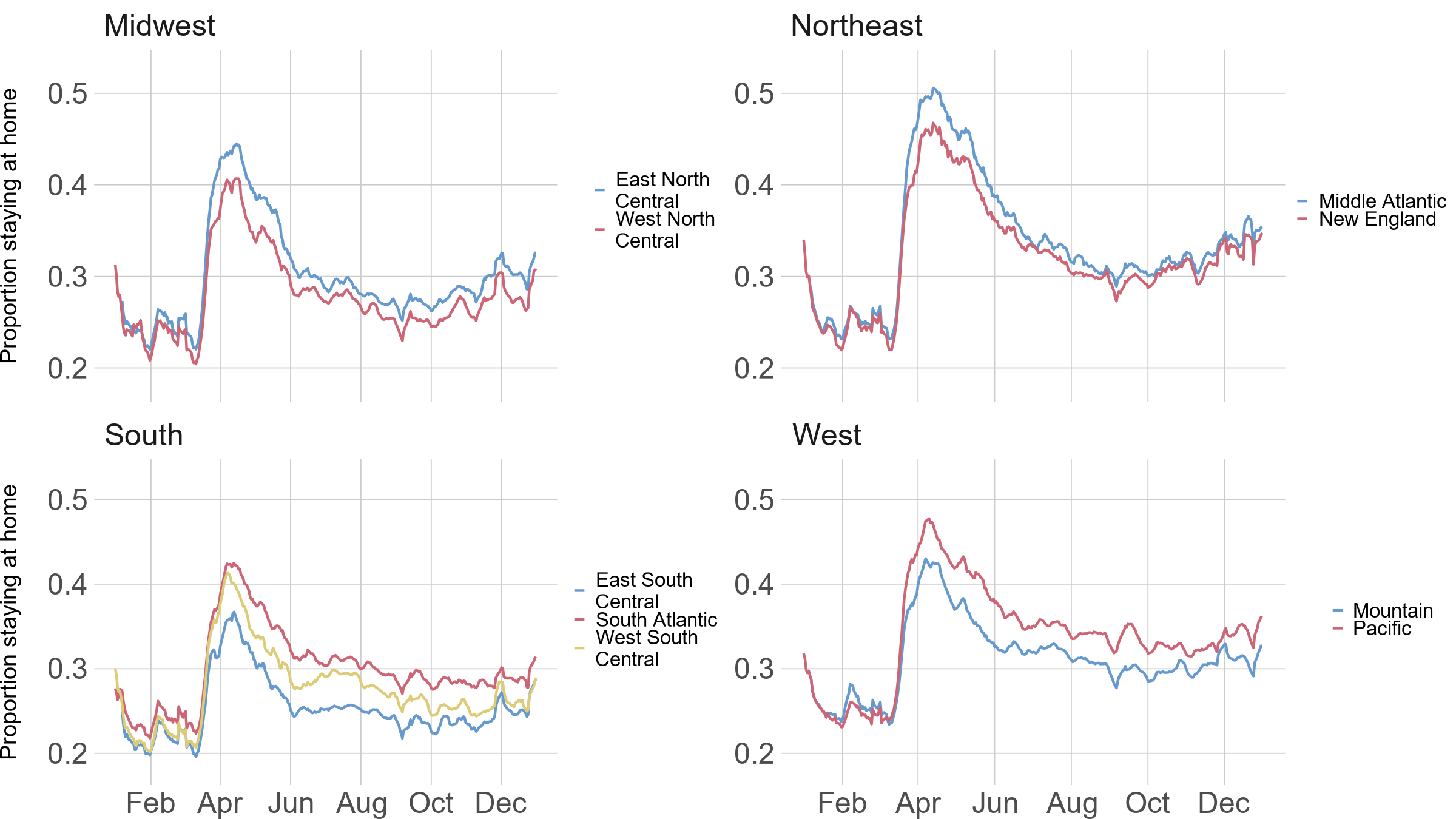

Supplement: S3 Fig — (TIFF) [file pone.0259665.s004.tiff]

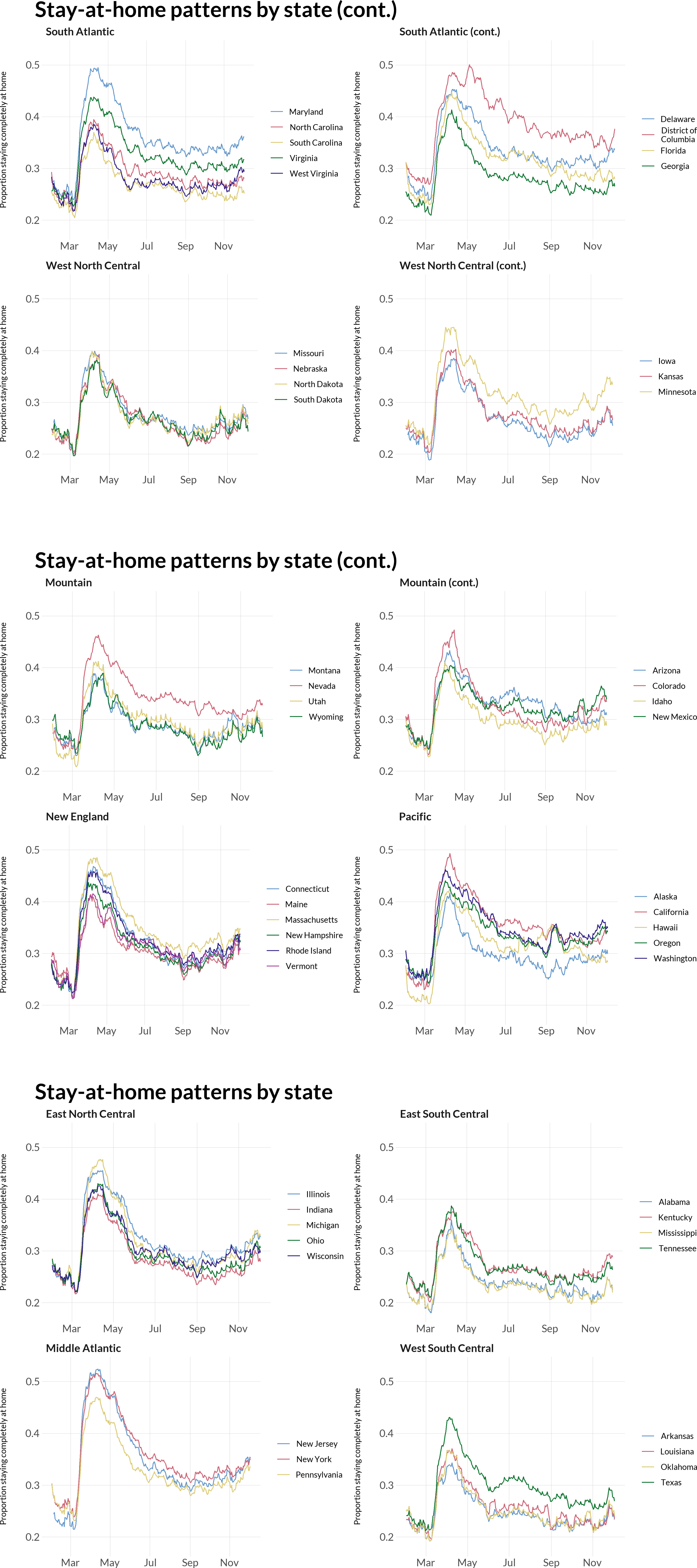

Supplement: S4 Fig — (A-C) Stay-at-home patterns by state. (PNG) [file pone.0259665.s005.png]

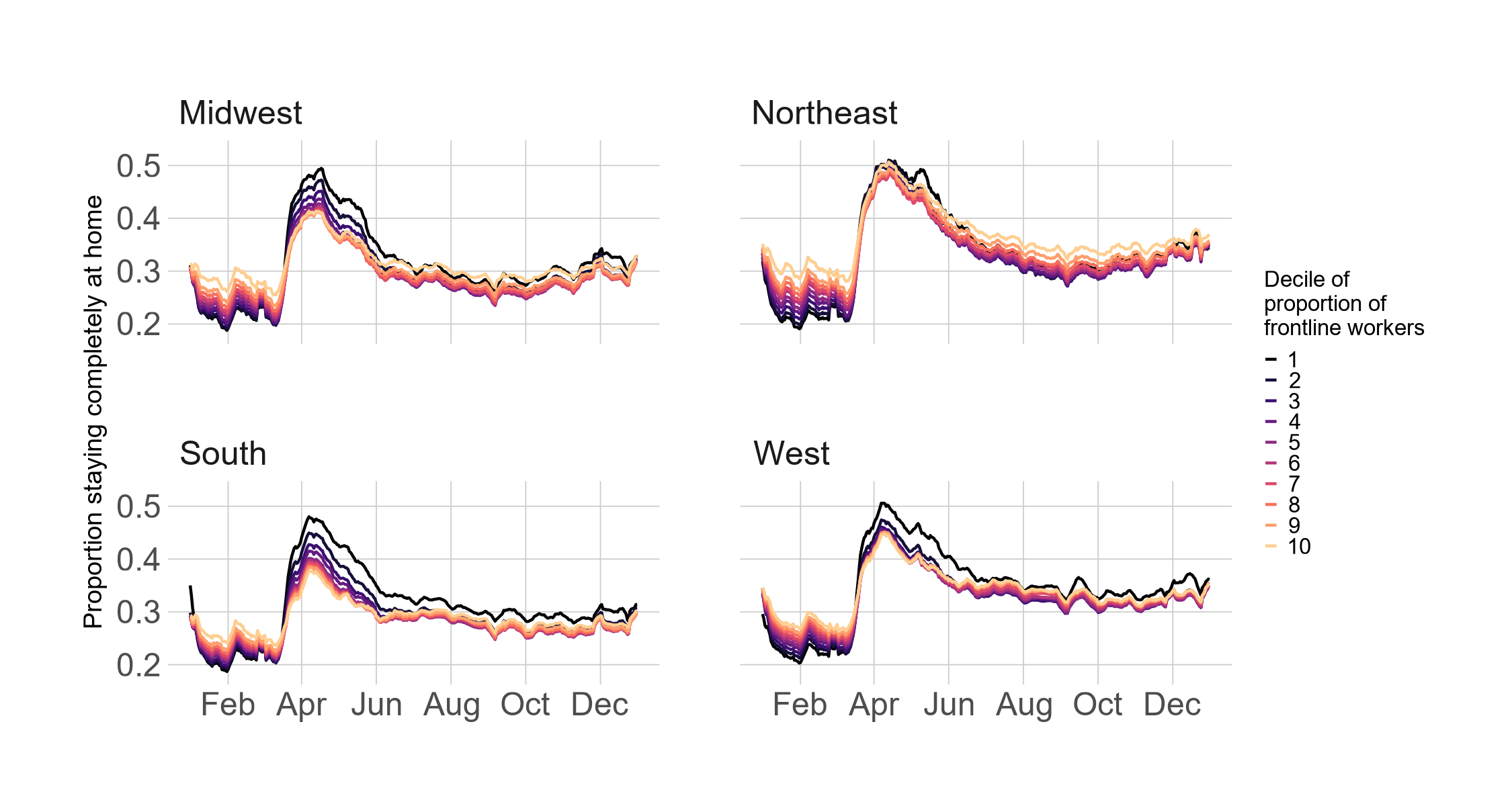

Supplement: S5 Fig — (TIFF) [file pone.0259665.s006.tiff]
