# Supplementary material for: U.S. regional differences in physical distancing: Evaluating racial and socioeconomic divides during the COVID-19 pandemic
Source: PLoS One. 2021 Nov 30;16(11):e0259665. doi: 10.1371/journal.pone.0259665 (PMC8631641; doi:10.1371/journal.pone.0259665)
Supplement: S1 Table — (DOCX) [file pone.0259665.s007.docx]

|  | | | | | | | |
| --- | --- | --- | --- | --- | --- | --- | --- |
|  | Midwest (N= 18,899,029, Adj R-squared= 0.19) | | |  | South (N= 26,923,163, Adj R-squared= 0.19) | | |
| Variable | Coefficient | SE | 95% CI |  | Coefficient | SE | 95% CI |
| Days from January 1st |  |  |  |  |  |  |  |
| Linear term | -2.33E-04 | 1.40E-06 | (-2.36E-04, -2.31E-04) |  | 1.29E-04 | 1.16E-06 | (1.27E-04,1.31E-04) |
| Quadratic term | 2.17E-07 | 2.98E-09 | (2.11E-07,2.22E-07) |  | -6.52E-07 | 2.47E-09 | (-6.57E-07, -6.47E-07) |
| Proportion Black | 0.076 | 1.53E-04 | (0.076,0.077) |  | 0.06 | 1.14E-04 | (0.060,0.061) |
| Period (Reference = Before April 1st) |  |  |  |  |  |  |  |
| April 1st-30th | 0.158 | 9.83E-05 | (0.158,0.158) |  | 0.139 | 8.83E-05 | (0.139,0.139) |
| After May 1st | 0.059 | 1.18E-04 | (0.059,0.059) |  | 0.047 | 9.98E-05 | (0.047,0.047) |
| Interaction between period and proportion Black |  |  |  |  |  |  |  |
| April 1st - April 30th * proportion Black | -0.049 | 3.00E-04 | (-0.050, -0.049) |  | -0.066 | 2.23E-04 | (-0.066, -0.065) |
| After May 1st * proportion Black | -0.003 | 1.78E-04 | (-0.003, -0.002) |  | -0.004 | 1.32E-04 | (-0.004, -0.004) |
| Intercept | 0.263 | 6.57E-05 | (0.263,0.263) |  | 0.231 | 5.72E-05 | (0.231,0.231) |
|  |  |  |  |  |  |  |  |
|  | Northeast (N= 15,035,157, Adj R-squared= 0.25) | | |  | West (N= 16,733,365, Adj R-squared= 0.23) | | |
|  | Coefficient | SE | 95% CI |  | Coefficient | SE | 95% CI |
| Days from January 1st |  |  |  |  |  |  |  |
| Linear term | -2.30E-04 | 1.80E-06 | (-2.33E-04, -2.26E-04) |  | 2.22E-04 | 1.57E-06 | (2.19E-04,2.25E-04) |
| Quadratic term | -1.89E-08 | 3.83E-09 | (-2.65E-08, -1.14E-08) |  | -8.87E-07 | 3.34E-09 | (-8.93E-07, -8.80E-07) |
| Proportion Black | 0.088 | 2.20E-04 | (0.087,0.088) |  | 0.089 | 4.86E-04 | (0.088,0.090) |
| Period (Reference = Before April 1st) |  |  |  |  |  |  |  |
| April 1st-30th | 0.212 | 1.28E-04 | (0.212,0.213) |  | 0.15 | 1.10E-04 | (0.150,0.151) |
| After May 1st | 0.102 | 1.52E-04 | (0.102,0.103) |  | 0.069 | 1.32E-04 | (0.068,0.069) |
| Interaction between period and proportion Black |  |  |  |  |  |  |  |
| April 1st - April 30th * proportion Black | -0.033 | 4.32E-04 | (-0.034, -0.032) |  | -0.002 | 9.51E-04 | (-0.004,0.000) |
| After May 1st * proportion Black | 0.006 | 2.56E-04 | (0.006,0.007) |  | 0.027 | 5.64E-04 | (0.025,0.028) |
| Intercept | 0.275 | 8.52E-05 | (0.275,0.275) |  | 0.261 | 7.37E-05 | (0.261,0.261) |
| Note: All p-values are smaller than 0.001. |  |  |  |  |  |  |  |
