# Supplementary material for: U.S. regional differences in physical distancing: Evaluating racial and socioeconomic divides during the COVID-19 pandemic
Source: PLoS One. 2021 Nov 30;16(11):e0259665. doi: 10.1371/journal.pone.0259665 (PMC8631641; doi:10.1371/journal.pone.0259665)
Supplement: S2 Table — (DOCX) [file pone.0259665.s008.docx]

|  | | | | | | | |
| --- | --- | --- | --- | --- | --- | --- | --- |
|  | Midwest (N= 18,897,847, Adj R-squared= 0.19) | | |  | South (N= 26,906,238, Adj R-squared= 0.17) | | |
| Variable | Coefficient | SE | 95% CI |  | Coefficient | SE | 95% CI |
| Days from January 1st |  |  |  |  |  |  |  |
| Linear term | -2.33E-04 | 1.42E-06 | (-2.36E-04, -2.30E-04) |  | 1.29E-04 | 1.17E-06 | (1.27E-04,1.32E-04) |
| Quadratic term | 2.16E-07 | 3.03E-09 | (2.10E-07,2.22E-07) |  | -6.53E-07 | 2.50E-09 | (-6.58E-07, -6.48E-07) |
| Proportion of frontline workers | 0.092 | 2.40E-04 | (0.091,0.092) |  | 0.054 | 1.95E-04 | (0.054,0.055) |
| Period (Reference = Before April 1st) |  |  |  |  |  |  |  |
| April 1st-30th | 0.225 | 1.93E-04 | (0.225,0.226) |  | 0.196 | 1.49E-04 | (0.196,0.197) |
| After May 1st | 0.088 | 1.55E-04 | (0.087,0.088) |  | 0.069 | 1.23E-04 | (0.069,0.069) |
| Interaction between period and proportion of Bachelor's degree holders |  |  |  |  |  |  |  |
| April 1st-30th * prop. frontline workers | -0.204 | 4.69E-04 | (-0.205, -0.203) |  | -0.21 | 3.82E-04 | (-0.211, -0.210) |
| After May 1st * prop. frontline workers | -0.081 | 2.78E-04 | (-0.082, -0.081) |  | -0.068 | 2.27E-04 | (-0.069, -0.068) |
| Intercept | 0.239 | 1.07E-04 | (0.239,0.240) |  | 0.225 | 8.40E-05 | (0.225,0.225) |
|  |  |  |  |  |  |  |  |
|  | Northeast (N= 15,027,003, Adj R-squared= 0.26) | | |  | West (N= 16,711,234, Adj R-squared= 0.22) | | |
|  | Coefficient | SE | 95% CI |  | Coefficient | SE | 95% CI |
| Days from January 1st |  |  |  |  |  |  |  |
| Linear term | -2.30E-04 | 1.82E-06 | (-2.33E-04, -2.26E-04) |  | 2.23E-04 | 1.57E-06 | (2.20E-04,2.26E-04) |
| Quadratic term | -1.90E-08 | 3.88E-09 | (-2.66E-08, -1.14E-08) |  | -8.89E-07 | 3.34E-09 | (-8.96E-07, -8.83E-07) |
| Proportion of frontline workers | 0.144 | 2.99E-04 | (0.143,0.145) |  | 0.09 | 2.75E-04 | (0.090,0.091) |
| Period (Reference = Before April 1st) |  |  |  |  |  |  |  |
| April 1st-30th | 0.26 | 2.17E-04 | (0.259,0.260) |  | 0.207 | 1.95E-04 | (0.207,0.208) |
| After May 1st | 0.13 | 1.85E-04 | (0.129,0.130) |  | 0.098 | 1.63E-04 | (0.098,0.099) |
| Interaction between period and proportion of Bachelor's degree holders |  |  |  |  |  |  |  |
| April 1st-30th * prop. frontline workers | -0.165 | 5.86E-04 | (-0.166, -0.164) |  | -0.184 | 5.38E-04 | (-0.185, -0.183) |
| After May 1st * prop. frontline workers | -0.085 | 3.48E-04 | (-0.086, -0.084) |  | -0.092 | 3.19E-04 | (-0.093, -0.091) |
| Intercept | 0.241 | 1.24E-04 | (0.240,0.241) |  | 0.237 | 1.11E-04 | (0.237,0.237) |
| Note: All p-values are smaller than 0.001. |  |  |  |  |  |  |  |
