# Supplementary material for: U.S. regional differences in physical distancing: Evaluating racial and socioeconomic divides during the COVID-19 pandemic
Source: PLoS One. 2021 Nov 30;16(11):e0259665. doi: 10.1371/journal.pone.0259665 (PMC8631641; doi:10.1371/journal.pone.0259665)
Supplement: S3 Table — (DOCX) [file pone.0259665.s009.docx]

|  | | | | | | | |
| --- | --- | --- | --- | --- | --- | --- | --- |
|  | Midwest (N= 18,903,105, Adj R-squared= 0.22) | | |  | South (N= 26,926,567, Adj R-squared= 0.19) | | |
| Variable | Coefficient | SE | 95% CI |  | Coefficient | SE | 95% CI |
| Days from January 1st |  |  |  |  |  |  |  |
| Linear term | -2.33E-04 | 1.42E-06 | (-2.36E-04, -2.31E-04) |  | 1.29E-04 | 1.16E-06 | (1.27E-04,1.31E-04) |
| Quadratic term | 2.17E-07 | 3.02E-09 | (2.11E-07,2.23E-07) |  | -6.52E-07 | 2.48E-09 | (-6.57E-07, -6.47E-07) |
| Proportion of Bachelor's degree holders | -0.064 | 1.90E-04 | (-0.064, -0.063) |  | -0.032 | 1.48E-04 | (-0.032, -0.032) |
| Period (Reference = Before April 1st) |  |  |  |  |  |  |  |
| April 1st-30th | 0.098 | 1.38E-04 | (0.097,0.098) |  | 0.072 | 1.09E-04 | (0.072,0.072) |
| After May 1st | 0.031 | 1.32E-04 | (0.031,0.032) |  | 0.024 | 1.07E-04 | (0.024,0.025) |
| Interaction between period and proportion of Bachelor's degree holders |  |  |  |  |  |  |  |
| April 1st-30th * prop. Bachelor's degree | 0.2 | 3.72E-04 | (0.199,0.201) |  | 0.201 | 2.90E-04 | (0.200,0.201) |
| After May 1st * prop. Bachelor's degree | 0.099 | 2.21E-04 | (0.098,0.099) |  | 0.08 | 1.72E-04 | (0.080,0.081) |
| Intercept | 0.29 | 8.24E-05 | (0.289,0.290) |  | 0.252 | 6.61E-05 | (0.252,0.252) |
|  |  |  |  |  |  |  |  |
|  | Northeast (N= 15,037,277, Adj R-squared= 0.28) | | |  | West (N= 16,736,238, Adj R-squared= 0.22) | | |
|  | Coefficient | SE | 95% CI |  | Coefficient | SE | 95% CI |
| Days from January 1st |  |  |  |  |  |  |  |
| Linear term | -2.30E-04 | 1.83E-06 | (-2.33E-04, -2.26E-04) |  | 2.22E-04 | 1.56E-06 | (2.19E-04,2.25E-04) |
| Quadratic term | -1.80E-08 | 3.89E-09 | (-2.56E-08, -1.04E-08) |  | -8.87E-07 | 3.32E-09 | (-8.93E-07, -8.80E-07) |
| Proportion of Bachelor's degree holders | -0.104 | 2.22E-04 | (-0.105, -0.104) |  | -0.074 | 1.92E-04 | (-0.074, -0.074) |
| Period (Reference = Before April 1st) |  |  |  |  |  |  |  |
| April 1st-30th | 0.156 | 1.93E-04 | (0.156,0.156) |  | 0.092 | 1.58E-04 | (0.092,0.093) |
| After May 1st | 0.071 | 1.76E-04 | (0.070,0.071) |  | 0.038 | 1.48E-04 | (0.037,0.038) |
| Interaction between period and proportion of Bachelor's degree holders |  |  |  |  |  |  |  |
| April 1st-30th * prop. Bachelor's degree | 0.151 | 4.34E-04 | (0.150,0.151) |  | 0.181 | 3.76E-04 | (0.180,0.182) |
| After May 1st * prop. Bachelor's degree | 0.093 | 2.57E-04 | (0.092,0.093) |  | 0.101 | 2.23E-04 | (0.100,0.101) |
| Intercept | 0.322 | 1.13E-04 | (0.321,0.322) |  | 0.289 | 9.36E-05 | (0.288,0.289) |
| Note: All p-values are smaller than 0.001. |  |  |  |  |  |  |  |
