# Supplementary material for: U.S. regional differences in physical distancing: Evaluating racial and socioeconomic divides during the COVID-19 pandemic
Source: PLoS One. 2021 Nov 30;16(11):e0259665. doi: 10.1371/journal.pone.0259665 (PMC8631641; doi:10.1371/journal.pone.0259665)
Supplement: S4 Table — (DOCX) [file pone.0259665.s010.docx]

|  | | | | | | | |
| --- | --- | --- | --- | --- | --- | --- | --- |
|  | Midwest (N= 18,500,754, Adj R-squared= 0.20) | | |  | South (N= 26,050,176, Adj R-squared= 0.19) | | |
| Variable | Coefficient | SE | 95% CI |  | Coefficient | SE | 95% CI |
| Days from January 1st |  |  |  |  |  |  |  |
| Linear term | -2.33E-04 | 1.42E-06 | (-2.36E-04, -2.30E-04) |  | 1.34E-04 | 1.18E-06 | (1.31E-04,1.36E-04) |
| Quadratic term | 2.17E-07 | 3.03E-09 | (2.11E-07,2.23E-07) |  | -6.62E-07 | 2.51E-09 | (-6.67E-07, -6.57E-07) |
| Income | -5.62E-04 | 1.24E-09 | (-5.64E-07, -5.59E-07) |  | 1.90E-04 | 5.28E-10 | (1.89E-07,1.91E-07) |
| Period (Reference = Before April 1st) | |  |  |  |  |  |  |
| April 1st-30th | 0.069 | 1.75E-04 | (0.069,0.070) |  | -0.019 | 1.16E-04 | (-0.020, -0.019) |
| After May 1st | 0.029 | 1.47E-04 | (0.029,0.029) |  | 0.031 | 1.18E-04 | (0.031,0.031) |
| Interaction between period and income | |  |  |  |  |  |  |
| April 1st-30th * income | 1.38E-03 | 2.43E-09 | (1.37E-06,1.38E-06) |  | -4.48E-04 | 1.04E-09 | (-4.50E-07, -4.46E-07) |
| After May 1st * income | 4.78E-04 | 1.44E-09 | (4.75E-07,4.81E-07) |  | 8.39E-04 | 1.59E-09 | (8.35E-07,8.42E-07) |
| Intercept | 0.306 | 9.93E-05 | (0.306,0.307) |  | 0.278 | 1.38E-04 | (0.278,0.278) |
|  |  |  |  |  |  |  |  |
|  | Northeast (N= 14,523,150, Adj R-squared= 0.27) | | |  | West (N= 16,349,784, Adj R-squared= 0.23) | | |
|  | Coefficient | SE | 95% CI |  | Coefficient | SE | 95% CI |
| Days from January 1st |  |  |  |  |  |  |  |
| Linear term | -2.31E-04 | 1.83E-06 | (-2.35E-04, -2.28E-04) |  | 2.25E-04 | 1.57E-06 | (2.22E-04,2.28E-04) |
| Quadratic term | -8.18E-09 | 3.91E-09 | (-1.58E-08, -5.18E-10) |  | -8.92E-07 | 3.34E-09 | (-8.99E-07, -8.86E-07) |
| Income | -6.29E-04 | 1.17E-09 | (-6.31E-07, -6.26E-07) |  | 1.01E-04 | 6.26E-10 | (1.00E-07,1.03E-07) |
| Period (Reference = Before April 1st) | |  |  |  |  |  |  |
| April 1st-30th | 0.135 | 2.11E-04 | (0.134,0.135) |  | -0.029 | 1.59E-04 | (-0.029, -0.028) |
| After May 1st | 0.072 | 1.84E-04 | (0.072,0.073) |  | 0.037 | 1.68E-04 | (0.037,0.037) |
| Interaction between period and income | |  |  |  |  |  |  |
| April 1st-30th * income | 9.89E-04 | 2.28E-09 | (9.84E-07,9.93E-07) |  | -5.59E-04 | 1.23E-09 | (-5.61E-07, -5.57E-07) |
| After May 1st * income | 3.99E-04 | 1.35E-09 | (3.97E-07,4.02E-07) |  | 5.99E-04 | 1.89E-09 | (5.95E-07,6.03E-07) |
| Intercept | 0.333 | 1.21E-04 | (0.332,0.333) |  | 0.327 | 1.85E-04 | (0.327,0.328) |
| Note: All p-values are smaller than 0.001. Median household income is in thousands of dollars. | | | | | |  |  |
