# Supplementary material for: U.S. regional differences in physical distancing: Evaluating racial and socioeconomic divides during the COVID-19 pandemic
Source: PLoS One. 2021 Nov 30;16(11):e0259665. doi: 10.1371/journal.pone.0259665 (PMC8631641; doi:10.1371/journal.pone.0259665)
Supplement: S5 Table — (DOCX) [file pone.0259665.s011.docx]

|  | | | | | | | |
| --- | --- | --- | --- | --- | --- | --- | --- |
|  | Midwest (N= 18,903,651, Adj R-squared= 0.18) | | |  | South (N= 26,926,487, Adj R-squared= 0.16) | | |
| Variable | Coefficient | SE | 95% CI |  | Coefficient | SE | 95% CI |
| Days from January 1st |  |  |  |  |  |  |  |
| Linear term | -2.33E-04 | 1.43E-06 | (-2.36E-04, -2.31E-04) |  | 1.29E-04 | 1.18E-06 | (1.27E-04, 1.31E-04) |
| Quadratic term | 2.16E-07 | 3.04E-09 | (2.10E-07, 2.22E-07) |  | -6.52E-07 | 2.51E-09 | (-6.57E-07, -6.47E-07) |
| Prop. of residents over the age of 65 | -0.024 | 4.20E-04 | (-0.024, -0.023) |  | 0.016 | 2.77E-04 | (0.016, 0.017) |
| Period (Reference = Before April 1st) | |  |  |  |  |  |  |
| April 1st-30th | 0.150 | 1.68E-04 | (.150, .150) |  | 0.132 | 1.21E-04 | (0.131, 0.132) |
| After May 1st | 0.063 | 1.44E-04 | (.063, .063) |  | 0.054 | 1.12E-04 | (0.053, 0.054) |
| Interaction between period and prop. residents over 65 | |  |  |  |  |  |  |
| April 1st-30th * prop. residents over 65 | 0.012 | 8.22E-04 | (0.011, 0.014) |  | -0.032 | 5.42E-04 | (-0.033, -0.031) |
| After May 1st * prop. residents over 65 | -0.026 | 4.87E-04 | (-0.027, -0.025) |  | -0.043 | 3.21E-04 | (-0.043, -0.042) |
| Intercept | 0.276 | 9.59E-05 | (0.276, 0.276) |  | 0.240 | 7.15E-05 | (0.240, 0.241) |
|  |  |  |  |  |  |  |  |
|  | Northeast (N= 15,038,243, Adj R-squared= 0.25) | | |  | West (N= 16,736,710, Adj R-squared= 0.21) | | |
|  | Coefficient | SE | 95% CI |  | Coefficient | SE | 95% CI |
| Days from January 1st |  |  |  |  |  |  |  |
| Linear term | -2.30E-04 | 1.83E-06 | (-2.33E-04, -2.26E-04) |  | 2.22E-04 | 1.57E-06 | (2.19E-04, 2.25E-04) |
| Quadratic term | -1.91E-08 | 3.91E-09 | (-2.67E-08, -1.14E-08) |  | -8.87E-07 | 3.36E-09 | (-8.94E-07, -8.80E-07) |
| Prop. of residents over the age of 65 | -0.045 | 4.94E-04 | (-0.046, -0.044) |  | 0.030 | 3.66E-04 | (0.029, 0.031) |
| Period (Reference = Before April 1st) | |  |  |  |  |  |  |
| April 1st-30th | 0.212 | 2.07E-04 | (0.211, 0.212) |  | 0.155 | 1.55E-04 | (0.154, 0.155) |
| After May 1st | 0.111 | 1.82E-04 | (0.110, 0.111) |  | 0.073 | 1.48E-04 | (0.072, 0.073) |
| Interaction between period and prop. residents over 65 | |  |  |  |  |  |  |
| April 1st-30th * prop. residents over 65 | -0.018 | 9.66E-04 | (-0.020, -0.016) |  | -0.027 | 7.16E-04 | (-0.028, -0.025) |
| After May 1st * prop. residents over 65 | -0.042 | 5.73E-04 | (-0.043, -0.041) |  | -0.017 | 4.24E-04 | (-0.017, -0.016) |
| Intercept | 0.293 | 1.19E-04 | (0.293, 0.293) |  | 0.260 | 9.26E-05 | (0.260, 0.260) |
| Note: All p-values are smaller than 0.001. | | | | | |  |  |
