# Supplementary material for: U.S. regional differences in physical distancing: Evaluating racial and socioeconomic divides during the COVID-19 pandemic
Source: PLoS One. 2021 Nov 30;16(11):e0259665. doi: 10.1371/journal.pone.0259665 (PMC8631641; doi:10.1371/journal.pone.0259665)
Supplement: Attachment — Submitted filename: response_to_reviewers_final.docx [file pone.0259665.s012.docx]

**Response Memo Detailing Revisions: PONE-D-21-13855 “U.S. Regional Differences in Physical Distancing: Evaluating Racial and Socioeconomic Divides During the COVID-19 Pandemic”**

We thank the editors and the reviewers for their careful review of our paper and useful suggestions for improvement. We have made revisions based on recommendations of the reviewers, and these recommendations have improved the paper. In this memo, we summarize, point by point, our revisions and responses to the reviewers’ comments. Comments from editors and reviewers are in **bold**.

**Journal Requirements:**

**1. Please ensure that your manuscript meets PLOS ONE's style requirements, including those for file naming. The PLOS ONE style templates can be found at**

[***https://journals.plos.org/plosone/s/file?id=wjVg/PLOSOne_formatting_sample_main_body.pdf***](https://journals.plos.org/plosone/s/file?id=wjVg/PLOSOne_formatting_sample_main_body.pdf) **and https://journals.plos.org/plosone/s/file?id=ba62/PLOSOne_formatting_sample_title_authors_affiliations.pdf**

We have carefully formatted all the files based on PLOS ONE’s style requirements.

**2. Please ensure that you include a title page within your main document. We do appreciate that you have a title page document uploaded as a separate file, however, as per our author guidelines (**[***http://journals.plos.org/plosone/s/submission-guidelines#loc-title-page***](http://journals.plos.org/plosone/s/submission-guidelines#loc-title-page)**) we do require this to be part of the manuscript file itself and not uploaded separately.
Could you therefore please include the title page into the beginning of your manuscript file itself, listing all authors and affiliations.**

We have included the title page into the beginning of our manuscript file.

**3. Please include your tables as part of your main manuscript and remove the individual files. Please note that supplementary tables should remain uploaded) as separate "supporting information" files.**

We have revised accordingly. Notably, our main table is best visible in “Draft” view in Word, but not in the “Print Layout” which Word often sets as the default.

**4. We note that you have indicated that data from this study are available upon request. PLOS only allows data to be available upon request if there are legal or ethical restrictions on sharing data publicly. For more information on unacceptable data access restrictions, please see** [***http://journals.plos.org/plosone/s/data-availability#loc-unacceptable-data-access-restrictions***](http://journals.plos.org/plosone/s/data-availability#loc-unacceptable-data-access-restrictions)**.**

**In your revised cover letter, please address the following prompts:**

**a) If there are ethical or legal restrictions on sharing a de-identified data set, please explain them in detail (e.g., data contain potentially sensitive information, data are owned by a third-party organization, etc.) and who has imposed them (e.g., an ethics committee). Please also provide contact information for a data access committee, ethics committee, or other institutional body to which data requests may be sent.**

**b) If there are no restrictions, please upload the minimal anonymized data set necessary to replicate your study findings as either Supporting Information files or to a stable, public repository and provide us with the relevant URLs, DOIs, or accession numbers. For a list of acceptable repositories, please see** [***http://journals.plos.org/plosone/s/data-availability#loc-recommended-repositories***](http://journals.plos.org/plosone/s/data-availability#loc-recommended-repositories)**.**

**We will update your Data Availability statement on your behalf to reflect the information you provide.**

We have explained our legal restrictions in the cover letter.

**5. We note that Figure Supporting Information ' 2020-04-01.png' in your submission contain map images which may be copyrighted. All PLOS content is published under the Creative Commons Attribution License (CC BY 4.0), which means that the manuscript, images, and Supporting Information files will be freely available online, and any third party is permitted to access, download, copy, distribute, and use these materials in any way, even commercially, with proper attribution. For these reasons, we cannot publish previously copyrighted maps or satellite images created using proprietary data, such as Google software (Google Maps, Street View, and Earth). For more information, see our copyright guidelines:** [***http://journals.plos.org/plosone/s/licenses-and-copyright***](http://journals.plos.org/plosone/s/licenses-and-copyright)**.**

**We require you to either (1) present written permission from the copyright holder to publish these figures specifically under the CC BY 4.0 license, or (2) remove the figures from your submission:**

**5.1.    You may seek permission from the original copyright holder of Figure Supporting Information ' 2020-04-01.png' to publish the content specifically under the CC BY 4.0 license. We recommend that you contact the original copyright holder with the Content Permission Form (**[***http://journals.plos.org/plosone/s/file?id=7c09/content-permission-form.pdf***](http://journals.plos.org/plosone/s/file?id=7c09/content-permission-form.pdf)**) and the following text: “I request permission for the open-access journal PLOS ONE to publish XXX under the Creative Commons Attribution License (CCAL) CC BY 4.0 (**[***http://creativecommons.org/licenses/by/4.0/***](http://creativecommons.org/licenses/by/4.0/)**). Please be aware that this license allows unrestricted use and distribution, even commercially, by third parties. Please reply and provide explicit written permission to publish XXX under a CC BY license and complete the attached form.” Please upload the completed Content Permission Form or other proof of granted permissions as an "Other" file with your submission.**

**In the figure caption of the copyrighted figure, please include the following text: “Reprinted from [ref] under a CC BY license, with permission from [name of publisher], original copyright [original copyright year].”**

**5.2.    If you are unable to obtain permission from the original copyright holder to publish these figures under the CC BY 4.0 license or if the copyright holder’s requirements are incompatible with the CC BY 4.0 license, please either i) remove the figure or ii) supply a replacement figure that complies with the CC BY 4.0 license. Please check copyright information on all replacement figures and update the figure caption with source information. If applicable, please specify in the figure caption text when a figure is similar but not identical to the original image and is therefore for illustrative purposes only.**

Our figures do not use any copyrighted geographic data; the shapefiles pertaining to the figure noted by the editor ('2020-04-01.png') come from the U.S. Census Bureau's 2019 iteration of the TIGER/Line program. These files are under the public domain and are copyright-free ([*https://www.usa.gov/government-works*](https://www.usa.gov/government-works)). This map also uses Safegraph social distancing data which are licensed, as noted in the cover letter, but as per Section 11d of the Safegraph Terms of Service are available for use to create content in academic publications. Because the data that are licensed under the Safegraph ToS are not downloadable or accessible by viewers of this figure, we consider the figure not copyrighted.

**6. Please include captions for your Supporting Information files at the end of your manuscript, and update any in-text citations to match accordingly. Please see our Supporting Information guidelines for more information: http://journals.plos.org/plosone/s/supporting-information.**

We have revised accordingly.

**Reviewer #1:**

**I am at a bit of a loss with this manuscript. You can measure physical distancing at the block group level, yet you content yourself with regional-level findings. On the one hand, this does little to build on the existing discussion as other research has already explored COVID-19 distancing behaviors by region. Gibbons (2021), for example, was just published in this journal on this question using the Google Mobility data. On the other hand, and maybe more importantly, this is a huge missed opportunity! The SafeGraph data offers the potential to address much more in-deph, and interesting, questions compared to what Gibbons and others have been able to do. The level of focus could be shifted to a smaller scale like metropolitan areas or some other smaller unit, which would offer much more richness.**

Thank you for the reference, which is highly relevant to our study. We have also added a couple of other relevant studies in the introduction that just came out after we submitted this manuscript. Based on our literature review, no studies have examined *Census regional* disparities in physical distancing patterns over time in the US during the pandemic. We have made it clearer in the manuscript that regional differences in this paper explicitly refer to differences across *Census regions*.

Gibbons (2021) mainly focused on racial/ethnic differences in social distancing patterns, and conducted in-depth analysis using data from Feb 16^th^ to March 29^th^, 2020. By contrast, our study focuses on Census regional disparities in physical distancing patterns, and examines the differential patterns by race and socioeconomic status within each region, using data throughout 2020. In addition, Gibbons (2021) used county-level data from Google Maps, whereas we used Census block group data from cell phone location pings. Because of the differences in aims, data sources, and units of analyses, our paper and Gibbons (2021) should be viewed as complements, not substitutes. We have discussed results from previous relevant studies more in the discussion section.

We agree that our data can be used to study many other important topics and can be used for more complicated analyses. But they should be separate studies, rather than this one, particularly considering it would be timely to have these descriptive results during the pandemic. We have further strengthened our rationale for studying regional disparities (page 4 second paragraph). Specifically, following a large number of studies examining health disparities across Census regions before the pandemic, we choose to focus on regions mainly for theoretical reasons: physical distancing may be *structurally constrained* by region. Understanding regional differences in physical distancing trends may highlight particular regions where COVID-19 mitigation policies and outreach should be targeted.

**Given the potential for spatial bias with such an ecological dataset, were spatial methods considered? At the very least, global and local Moran’s I tests should be conducted on the outcome measures to see if they suffer from any underlying spatial autocorrelation. If autocorrelation exists, more advance spatial modelling that accounts for spatial lag or error should be considered.**

As mentioned in the paper, the purpose of the study is to present descriptive patterns of regional variation in physical distancing over time rather than examining causal determinants of these behaviors (page 6 last paragraph). Therefore, examining whether the physical distancing patterns of one place is determined by the patterns of places nearby or some other factors is beyond the scope of the study. In addition, Moran’s I tests rely on both an index and a p-value. With a sample size as large as ours, the p-value is guaranteed to be significant. We have added a sentence in the discussion section, highlighting the need to adopt advanced spatial modelling strategies in future studies on examining the determinants of social distancing (page 18 second paragraph).

**There are some other predictors that should be added to the models, including population density, transit accessibility (number of commuters, commute time, car users/public transit users, etc). These kinds of measures will help account for the need and ability of people to avoid work and other places.**

As mentioned in the paper, the purpose of the study is to present descriptive patterns of regional variation in physical distancing over time and examine differences within each region by race and SES, rather than examine causal determinants of these behaviors (page 6 last paragraph). Therefore, we choose to focus on race/ethnicity and SES, as well as two basic demographic covariates (age composition and urbanicity). Our focus on race/ethnicity and SES is driven by existing literature documenting that these two are the most important dimensions along which health disparities are shaped. Our main results are the visualizations, and the regression results are just complementary, which confirm the patterns in the figures. In addition, we believe our results are timely and could be useful for policymaking during the pandemic with a speedy publication. For all these reasons, systematically examining the causal determinants of physical distancing patterns should be a separate paper. In response to your request, we have added a sentence in the discussion section, highlighting the need to examine the causal determinants of social distancing, including population density and transit accessibility (page 18 second paragraph).

**Reviewer #2: -The manuscript topic is interesting and timely.**

Thank you! **-To improve this manuscript:

A-The authors need to do more analysis and explanation for the their study.**

We have 1) further strengthened our rationale for studying regional differences in the introduction, 2) expanded our explanation of the results in the discussion section, 3) discussed more relevant previous studies and spelled out how our analyses are different.

We are not completely sure what additional analyses you are referring to. We already included a large number of analyses (see figures, tables, and appendices). For our research aims, which is to present descriptive patterns of regional variation in physical distancing over time rather than examining causal determinants of these behaviors (page 6 last paragraph), we believe our analyses are sufficient. We are happy to add more if you have specific suggestions.
 **B-The authors need to explain how this study is different and unique from the other studies?**

From our literature review, we have not seen any studies examining Census regional differences in physical distancing patterns during the COVID-19 pandemic in the US. We have added a couple of additional relevant studies that just came out since we submitted our paper. We have made our unique contributions clearer in the introduction and discussion sections (pages 5-6).

**C- The Manuscript writing language needs to be edited and improved.**

We have carefully edited the manuscript and improved writing.
