# Supplementary material for: U.S. regional differences in physical distancing: Evaluating racial and socioeconomic divides during the COVID-19 pandemic
Source: PLoS One. 2021 Nov 30;16(11):e0259665. doi: 10.1371/journal.pone.0259665 (PMC8631641; doi:10.1371/journal.pone.0259665)
Supplement: Attachment — Submitted filename: response_to_reviewers.docx [file pone.0259665.s013.docx]

**Response Memo Detailing Revisions: PONE-D-21-13855 “U.S. Regional Differences in Physical Distancing: Evaluating Racial and Socioeconomic Divides During the COVID-19 Pandemic”**

We thank the editors and the reviewers for their careful review of our paper and useful suggestions for improvement. We have made revisions based on recommendations of the reviewers, and these recommendations have improved the paper. In this memo, we summarize, point by point, our revisions and responses to the reviewers’ comments. Comments from editors and reviewers are in **bold**.

**Reviewer #1:**

**The explanation that your effort is ‘descriptive’ instead of ‘causal’ is not sufficient to justify your modelling choices. You are still using inferential models which are subject to spatial bias and underestimation due to the lack of all needed predictors. Moran’s I test should be done and spatial modelling done depending on those results. Even with a large dataset like yours, this should be doable on R. Also, you should include population density and some measure of accessibility.**

Thank you for the comment, and we agree with your point on spatial bias and the existence of potential confounders. However, even with spatial models and indicators of population density and accessibility, there are still confounders which prohibit us from making causal claims. For our research question, which is related to “what is going on” rather than “why this is going on”, we are not sure trying to make a causal claim is necessary, and, without an experimental design, we are not sure establishing a causal claim is even possible in this case.

In our opinion, the advantage of a dataset as large as ours is its ability to provide rich details on the descriptive patterns. Its advantages on establishing causal claims are unclear. Therefore, as mentioned in the previous response and in the paper, the purpose of the study is to present descriptive patterns of regional variation in physical distancing *over time* (page 6 last paragraph), and our main results are *the visualizations*, with complementary regression results confirming the patterns in the figures.

In response to your request, we have added indicators of population density and average commute time as additional controls in our model (Table 1), and the results hardly changed. We have also tried to conduct spatial analyses. However, most previous studies have only used data for a couple of months, whereas we have data for the full year of 2020. Even using our high-speed computing cluster, we were not able to conduct analyses using the full sample due to the extremely large sample size and the complexity of the data. We have also experimented with multiple R packages and Python, but none of these worked.

In response to your request, though, we picked some random days throughout the year and conducted Moran’s I test and spatial analyses on those days. See the results below. The results for Moran’s I test unsurprisingly show the existence of spatial correlations. The results from spatial models show consistent patterns as those in our figures. Due to page limits, we did not add these results to the paper but would be happy to include them in appendix if needed.

Here are some details of the spatial models we ran. To account for potential spillover effects between block groups in close geographic proximity, in which the rates of physical distancing in any given block group is related not only to its own socioeconomic characteristics but the physical distancing pattern of its neighbor, we fit a set of two-staged least squares models that account for spatial lag in their dependent variable. These models are of the form:

y = ρWy + Xβ + ε

Where y is our outcome variable (the proportion staying at home), ρ represents the degree of spatial autocorrelation present and ranges from -1 to 1 (with 0 being no autocorrelation present), W is a spatial weights matrix, X being the matrix of independent variables, β being a vector of their associated coefficients representing their relationship with y, and ε representing the error term. "Spatial weights matrix" (or W here) represents connectedness between all possible pairs of block groups. A cell at column c and row r contains a 1 if the block group represented by c and the block group represented by r have at least one coordinate intersecting, and the cell contains 0 otherwise.

Detailed introduction of this model and the comparison between this and other spatial models can be found in the references below:

Anselin, L., & Smirnov, O. (1996). Efficient algorithms for constructing proper higher order spatial lag operators. Journal of regional science, 36(1), 67-89.

Anselin, Luc. "Spatial econometrics." A companion to theoretical econometrics 310330 (2001).

Cliff, A. D., Ord, J. K. 1981 Spatial processes, Pion.

Kelejian, H.H. and I.R. Prucha (1998). A generalized spatial two stage least squares procedure for estimating a spatial autoregressive model with autoregressive disturbances. Journal of Real Estate Finance and Economics 17, 99-121.

Roger Bivand, Gianfranco Piras (2015). Comparing Implementations of Estimation Methods for Spatial Econometrics. Journal of Statistical Software, 63(18), 1-36. [*https://www.jstatsoft.org/v63/i18/*](https://www.jstatsoft.org/v63/i18/).

| Moran's I test for spatial autocorrelation | | | | |
| --- | --- | --- | --- | --- |
|  |  |  |  |  |
| **Date** | **Moran's I statistic** | **Expectation** | **Variance** | ***p*-value** |
| Feb 15, 2020 | 0.278 | -4.06E-06 | 1.53E-06 | < 2.2e-16 |
| April 15, 2020 | 0.515 | -4.61E-06 | 1.54E-06 | < 2.2e-16 |
| Aug 15, 2020 | 0.268 | -4.61E-06 | 1.54E-06 | < 2.2e-16 |

| Regional disparities in physical distancing, accounting for spatial spillover between block groups | | | |
| --- | --- | --- | --- |
|  | 2020-02-15 | 2020-04-15 | 2020-08-15 |
| Region |  |  |  |
| *Northeast* | -0.007*** | 0.031*** | 0.026*** |
|  | (0.001) | (0.003) | (0.003) |
| *South* | -0.025*** | -0.017*** | 0.005*** |
|  | (0.002) | (0.001) | (0.001) |
| *West* | -0.006*** | 0.008*** | 0.044*** |
|  | (0.001) | (0.001) | (0.004) |
| Rho | -0.249** | 0.518*** | 0.192** |
|  | (0.106) | (0.041) | (0.080) |
| Intercept | 0.322*** | 0.205*** | 0.220*** |
|  | (0.027) | (0.017) | (0.022) |
| *N* | 214,473 | 214,473 | 214,473 |

| Regional disparities in physical distancing, accounting for spatial spillovers and socioeconomic covariates | | | |
| --- | --- | --- | --- |
|  | February 15th, 2020 | April 15th, 2020 | August 15th, 2020 |
| Region |  |  |  |
| *Northeast* | 0.002*** | 0.004*** | 0.004*** |
|  | (3.629x10^-04) | (0.001) | (0.001) |
| *South* | -0.015*** | -0.008*** | -0.001 |
|  | (3.615x10^-04) | (4.630x10^-04) | (0.001) |
| *West* | 0.004*** | -0.002*** | 0.010*** |
|  | (3.494x10^-04) | (4.421x10^-04) | (0.001) |
| Urbanicity (urban = 1) | 0.011*** | 0.011*** | 0.005*** |
|  | (3.579x10^-04) | (0.001) | (0.001) |
| Median income | -3.915x10^-07*** | 9.755x10^-08*** | -1.816x10^-07*** |
|  | (5.577x10^-09) | (6.413x10^-09) | (8.695x10^-09) |
| Proportion Black | 0.032*** | 0.006*** | 0.015*** |
|  | (0.001) | (0.001) | (0.001) |
| Proportion of frontline workers | 0.024*** | 0.002 | 0.009*** |
|  | (0.001) | (0.001) | (0.002) |
| Proportion with a Bachelor's degree | -0.012*** | 0.017*** | 0.017*** |
|  | (0.001) | (0.001) | (0.002) |
| Proportion over the age of 65 | 0.041*** | 0.020*** | 0.015*** |
|  | (0.001) | (0.001) | (0.002) |
| Commute time in minutes | 1.123x10^-04*** | 0.001*** | 3.224x10^-04*** |
|  | (1.629x10^-05) | (2.733x10^-05) | (3.310x10^-05) |
| Density, population per square km. | -2.420x10^-11 | 2.509x10^-12 | -8.860x10^-12 |
|  | (2.460x10^-11) | (3.112x10^-11) | (4.175x10^-11) |
| Rho | 0.405*** | 0.821*** | 0.837*** |
|  | (0.008) | (0.007) | (0.014) |
| Intercept | 0.150*** | 0.039*** | 0.032*** |
|  | (0.002) | (0.002) | (0.003) |
| *N* | 214,473 | 214,473 | 214,473 |

Finally, we have acknowledged the limitation of spatial bias and other potential confounders in our analyses (page 21 second paragraph).  **Also, I am well aware of the geography in which you are focused. My reservation is what is to be gained by such a large-scaled focus when this data is capable of so much more. As it stands, that is not enough of a reason to give a negative recommendation, however, more should be done to sell the focus on such a scale. Why should we care about census regions when so much of the variation is happening within these regions?**

Thank you for the comment. We agree that our data are rich and can be used for many other topics. However, our choice of research question is not driven by the data. On the contrary, our choice of the data is driven by the research question, which is important to study based on existing sociological/epidemiological theories and empirical evidence. We have spent a lot of space explaining why examining Census regions is important on pages 3-5 lines 55-94, page 6 lines 127-129.

Here are some additional thoughts. Due to page limits, we did not add these in the paper but would be happy to add if needed.

Research on geographic variation in health in the U.S. struggles with a lack of consistent definition of said geographies. The use of different measures of geographic location across studies prevents us from understanding the mechanisms that produce these disparities in health. For example, consider research by Montez and colleagues (2020) which shows variation in life expectancy. Specifically, states that adopted more liberal policies from 1970-2014 were shown to experience an increase in life expectancy. In contrast, states that adopted more conservative policies experienced a decline in life expectancy. While informative, it is not necessarily clear whether state or conservative policies are the mechanism driving the resulting disparity.

Much research uses cultural factors to explain geographic variation in health, particularly exercise, diet, and the consumption of alcohol and tobacco (Howard & Howard, 2020; Park et al., 2011). Such cultural factors impact health in a manner that is broader than legal or political boundaries. For example, the “Southern diet” has been defined in public health research as one that is high in sugary beverages, fried food, fats, and eggs/processed meats (Shikany et al., 2018). Cultural norms transcend county and state boundaries to impact the health of entire regions.

Howard, G., & Howard, V. J. (2020). Twenty Years of Progress Toward Understanding the Stroke Belt. *Stroke, 51*(3), 742-750.

Montez, J. K., Beckfield, J., Cooney, J. K., Grumbach, J. M., Hayward, M. D., Koytak, H. Z., et al. (2020). US State Policies, Politics, and Life Expectancy. *The Milbank Quarterly*, 1-32.

Park, K., Rimm, E., Siscovick, D., Spiegelman, D., Morris, J. S., & Mozaffarian, D. (2011). Demographic and lifestyle factors and selenium levels in men and women in the U.S. *nrp, 5*(4), 357-364.

Shikany, J. M., Safford, M. M., Bryan, J., Newby, P. K., Richman, J. S., Durant, Raegan W., et al. (2018). Dietary Patterns and Mediterranean Diet Score and Hazard of Recurrent Coronary Heart Disease Events and All‐Cause Mortality in the REGARDS Study. *Journal of the American Heart Association, 7*(14), e008078.
